# ARID1A downregulation promotes cell proliferation and migration of colon cancer via VIM activation and CDH1 suppression

**DOI:** 10.1111/jcmm.17590

**Published:** 2022-11-24

**Authors:** Salem Baldi, Qianshi Zhang, Zhenyu Zhang, Mohammed Safi, Hassan Khamgan, Han Wu, Mengyan Zhang, Yuanyuan Qian, Yina Gao, Abdullah Shopit, Abdullah Al‐Danakh, Mohammed Alradhi, Murad Al‐Nusaif, Yunfei Zuo

**Affiliations:** ^1^ Department of Clinical Biochemistry, College of Laboratory Diagnostic Medicine Dalian Medical University Dalian China; ^2^ Departments of Gastrointestinal Surgery The Second Affiliated Hospital of Dalian Medical University Dalian China; ^3^ Department of respiratory, Shandong Second Provincial General Hospital Shandong University Jinan China; ^4^ Department of Molecular Diagnostics and Therapeutics, Genetic Engineering & Biotechnology Research Institute (GEBRI) University of Sadat City Sadat Egypt; ^5^ Department of Pharmacology, School of Pharmacy, Academic Integrated Medicine & Collage of Pharmacy Dalian Medical University Dalian China; ^6^ Department of Urology First Affiliated Hospital of Dalian Medical University Dalian China; ^7^ Department of Urology the Affiliated Hospital of Qingdao Binhai University Qingdao China; ^8^ Department of Neurology First Affiliated Hospital of Dalian Medical University Dalian China

**Keywords:** ARID1A knockdown, CDH1, colon cancer, invasion, migration, VIM

## Abstract

According to our prior findings, ARID1A expression is decreased in colon cancer, which has a poor prognosis. In this study, we investigated the ARID1A‐VIM/CDH1 signalling axis's role in colon cancer proliferation and migration. The differentially expressed genes in cells that might be controlled by ARID1A were discovered by a database screening for ARID1A knockout. qPCR was used to analyse ARID1A and EMT markers expression levels in colon cancer. We utilized siRNA RID1A to explore the influence of ARID1A silencing on EMT in CRC cells. The function of ARID1A in the colon was investigated utilizing the wound healing, transwell and CCK‐8 WST‐ assays. The molecular mechanism by which ARID1A regulates VIM and CDH1 was elucidated using chip‐qPCR. Numerous genes involved in EMT were dysregulated in the absence of ARID1A. VIM expression increased in cells lacking ARID1A expression and vice versa. Many COAD samples with high ARID1A mRNA expression had low VIM mRNA expression, despite the relevance. CDH1 gene was positively correlated with ARID1A. Moreover, siRNA‐ARID1A‐transfected cells accelerated cell migration and invasion and increased cell proliferation rate in vitro. Chip‐qPCR analysis showed that ARID1A binds to the promoters of both genes and changes their expression in colon cancer. ARID1A inactivation is associated with VIM activation and CDH1 suppression, which might serve as crucial molecules influencing COAD prognosis, accelerate tumour progression, and shorten patients' survival time, and promote metastases of COAD. Thus, depletion of ARID1A can be therapeutically exploited by targeting downstream effects to improve cancer treatment‐related outcomes.

## 1 INTRODUCTION

Mutations in epigenetic regulators, particularly chromatin remodelling proteins and histone modifiers, have lately been found in cancer patients' genomes.^1^, ^2^ Nucleosome remodelling and transcriptional regulation are carried out by the SWI/SNF chromatin remodelling complex in an ATP‐dependent manner.^3^ The two major forms of this complex are BAF, a, BRG1‐associated factor and polybromo BAF2. Dysfunction of these subunits has been connected to abnormal control of lineage‐specific differentiation and gene expression/repression regulation, contributing to tumour development.^4^ Next‐generation sequencing identified AT‐rich interactive domain 1A (ARID1A) as the most frequently mutated BAF complex driver gene in the majority of cancer types, including colorectal cancer (CRC).^4^ It was reported that ARID1A, which functions as a transcription factor (TF), is associated with the chromatin state and binds promoter regions of transcription factors in a sequence‐specific manner to drive SWI/SNF recruitment.^5^ Furthermore, ARID1A has been involved in various protein–protein interaction and regulation mechanisms.^6^ Mutations in the ARID1A gene are frequently nonsense, which is consistent with a suppressor role for ARID1A in various cancers.^7^, ^8^ Moreover, loss of ARID1A expression has been found in a variety of malignancies, including breast and renal cancer.^9^ We and others found that loss of ARID1A expression was highly related to tumour development and poor prognosis in colon adenocarcinoma (COAD).^10^, ^11^, ^12^, ^13^ Epithelial–mesenchymal transition (EMT) is crucial in cancer growth, invasion and metastasis.^14^, ^15^ In a study published in 2019, the authors found that ARID1A deletion, which promotes the migration of aberrant endometrial tissue, increases EMT‐related gene expression.^7^ WANG et al. also reported that ARID1A influences the migration and invasion of breast cancer cells via EMT.^16^ Other studies predicted that ARID1A is a potential prognostic indicator for hepatocellular carcinoma, possibly through promoting EMT,^17^ and that loss of expression was linked to pancreatic neuroendocrine tumour metastasis and reduced patient survival time.^18^ However, the molecular mechanisms by which ARID1A is involved in colon cancer metastasis and primary tumorigenesis are not well understood. Nevertheless, the correlation between ARID1A expression and EMT‐associated markers, such as Vimentin and E‐cadherin expression in COAD, has not been reported. Our study focused on the specific molecular mechanism of ARID1A in colon cancer proliferation and metastasis via VIM/CDH dysregulation. This paper discovered the important role of ARID1A in colon cancer and proved that ARID1A directly targets VIM/CDH1 to regulate the proliferation and migration of colon cancer.

## 2 MATERIALS AND METHODS

### 2.1 Differentially Expressed Genes (DEGs) in ARID1A‐KO vs. WT

To investigate the expression of metastase ARID1A KO‐related genes, we used the GEO accession number GSE122926 database to identify genes with differential expression (DEGs) between HCT‐116‐ARID1A‐WT and HCT‐116‐Knockout. Genes were classified as downregulated or upregulated according to *p* < .05 and log2 (Change‐in‐Fold) > 1.0 or < −1.0, respectively.^19^

### 2.2 Gene expression and KEGG pathways correlation

Genome‐wide gene set variation analysis was performed on patients with colon cancer to determine the expression of pathways. The HOME‐For‐Researchers database (http://www.home‐for‐researchers.com/static/index.html#/) was used to co‐analyse gene expression and KEGG pathways using Pearson analysis.

### 2.3 Kaplan–Meier survival analysis and prognostic significance

To determine the prognostic significance of ARID1A‐KO‐related genes and different pathways in COAD, the DEGs included in the analysis results were filtered and evaluated for prognostic value using the Kaplan–Meier method provided in the Home‐for‐researchers payable online analysis tool. Additionally, GSCA (gene set cancer analysis) http://bioinfo.life.hust.edu.cn/GSCA/#/ was applied to find the pathway activity and expression stages whose expression levels had an impact on COAD patients (GSCA: Gene Set Cancer Analysis (hust.edu.cn) GEPIA2 (gepia2.cancer‐pku.cn)) is a publicly accessible portal that contains information on all 32 cancer types included in the TCGA. It provides biologists and clinicians with a one‐of‐a‐kind tool for accessing, comparing and analysing cancer multi‐omics data. We used GEPIA2 to determine the association between ARID1A gene and EMT marker gene expression in COAD.

### 2.4 GEPIA

GEPIA2 (http://gepia2.cancer‐pku.cn/#index) is a publicly accessible portal that contains information on all 32 cancer types included in the TCGA. It provides biologists and clinicians with a one‐of‐a‐kind tool for accessing, comparing and analysing cancer multi‐omics data.^20^ The GEPIA2 gene expression tool was used to assess the levels of VIM and CDH1 expression, study the prognosis of ARID1A in COAD, and examine the relationship between the ARID1A gene and the manifestation of EMT marker genes in COAD.

### 2.5 Tissue samples

A total of 29 paired and matched non‐cancerous colon tissues from patients undergoing surgery at the Second Affiliated Hospital of Dalian Medical University (DMU) have been collected. The written consent of all patients was collected, and the Clinical Research Ethics Committee of the Second Hospital of DMU University authorized the study. The tissues were frozen at −80°C until RNA extraction was conducted using the triazol reagent.

### 2.6 Cell culture and transfection

Briefly, the cells were seeded in a 12‐well plate and grown in an antibiotic‐free growth medium containing 10% FBS overnight. According to the kit instruction, the minimum final concentration of RNAi oligo was 50 nM. Briefly, in each well, 4 μl of siARID1A at an indicated concentration suspended in serum‐free was mixed with 100 μl serum‐free medium containing 4 μl siRNA Transfection Reagent (lipofectamin 2000, Gene pharma) and the transfection mixture was incubated at 37°C for 20 min.

After that, siARID1A or control was applied, and the cells were incubated for 4–6 h at 37°C in a humidified incubator with 5% CO_2_. The cells were then cultured in a complete growth medium for 24 and 48 h before digestion.

### 2.7 Qualitative real‐time PCR

The HCT116 colorectal cells expressing ARID1A wild type (ARID1A+/+) were transfected with siRNA targeting the ARID1A gene and total RNA was extracted with Trizole reagent according to the manufacturer's instructions. cDNA synthesis was performed according to the manufacturer's instructions using M‐MLV reverse transcriptase, RNase inhibitor and the resultant cDNA was then subjected to real‐time quantitative PCR to determine the relative mRNA levels of ARID1A normalized to the GAPDH value. The quantitative PCR using SyberGreen (was performed as follows, 95°C for 10 min; 40 cycles of 95°C for 30 s, 58°C for 20 s, and 72°C for 15 s; melting curve from 60 to 95°C, increasing in increments of 0.5°C every 5 s). The experiments were conducted in triplicate to validate the results. The human primers and sequence are listed in Table 1.

**TABLE 1:** Primer lists and their sequences used in the study

Gene name	Sequence (5’to 3′)
SiRNA‐ARID1A	S: GCCCUGAACAAUAACCUCATT AS: UGAGGUUAUUGUUCAGGGCTT
ARID1A negative control	S: UUCUCCGAACGUGUCACGUTT AS: ACGUGACACGUUCGGAGAATT
ARID1A F ARID1A R	TTATGACAGAGTGAGGACGGAG TGCCTTGGGTGGAGAACTGAT
CDH1 F CDH R	CGAGAGCTACACGTTCACGG GGGTGTCGAGGGAAAAATAGG.
VIM F VIM R	GACGCCATCAACACCGAGTT CTTTGTCGTTGGTTAGCTGGT

Abbreviations: S, sense; AS, antisense; F, forward; R, reverse.

### 2.8 Western blotting

Using RIPA lysis solution with PMSF and protease inhibitors, total protein was extracted from the cells, and the BCA method in lysis buffer was used to determine its concentration (Beijing Solaribo Science & Technology). Total protein was separated by SDS‐PAGE and transferred to a Millipore PVDF membrane using 6 or 8% of the total protein concentration. The membrane was incubated overnight at 4°C with rabbit antibodies against β‐actin (1:1000, Beijing Zhongshan Biotechnology Co., Ltd); ARID1A (1:300, CUSABIO TECHNOLOGY LLC); E‐cadherin Polyclonal antibody, cat on: 20874‐1‐AP; and Vimentin Polyclonal antibody, cat no: 10366‐1‐AP after blocking with 5% non‐fat milk in Tris‐buffered saline containing 0.05%Tween‐20 (1–1000). After washing, the membrane was incubated with a secondary antibody conjugated to horseradish peroxidase for 2 h at 37°C (Beijing Solaribo Science & Technology).

### 2.9 Cell scratch test

The cells were briefly implanted in a 12‐well plate with a complete medium of growth until they came together. Then, the cell monolayers were scratched using a 200‐ml pipette along the cultural wave diameter to generate a cell‐free region. The cells were further retained in a humidified incubator with % CO2 at 37°C following washing with PBS to remove unattached cells and debris. The scratched wound size was measured at certain time intervals (0, 24, and 48 h).

### 2.10 Transwell migration experiment

A haemocytometer was used to count cells that had been transfected with siRNA‐ARID1A for 24 h before digestion, centrifugation and resuspended in serum‐free media. 500 ml of serum‐containing media filled the Transwell plate's lower chamber. The upper chamber was filled with 200 ml of serum‐free medium containing cells.

After 24 h of growth, the medium was discarded, and the cells were fixed in 80 percent ethanol for 15 min. For 30 min, crystal violet solution (0.5 percent) was used to stain the cells. The upper chambers containing nonmigrated cells were gently wiped with a cotton swab and dried at room temperature after being washed twice with PBS. An Olympus microscope was used to capture the images, which were then counted using image software.

### 2.11 Cell counting and proliferation (CCK8)

We used the CCK‐8 kit (APExBIO, Shanghai, China) to conduct an ELISA to measure cell proliferation rate. ARID1A‐si and NC groups were divided into subgroups. After transfecting the cells (HCT116) for 48 h, 100 μl of cell suspension was used to seed each group at a density of 5000 cells/well. The cells were incubated for 24 h, 48 h and 72 h, then the CCK‐8 kit solution was added to each well and incubated for an additional 3 h. The Microplate spectrophotometer was used to detect the absorbance at 450 nm 3 h after adding the kit solution. There were three iterations of the experiments.

### 2.12 Co‐immunoprecipitation for protein–protein interaction

For the study of protein–protein interactions, co‐immunoprecipitation (co‐IP) of protein complexes from cell lysates is a commonly used technique. In‐gel digestion technique is a classical sample preparation method for mass spectrometry‐based proteomics that helps increase the results' reliability and reproducibility. The detection of ARID1A‐bound proteins in HCT116 was performed using an optimized classical co‐IP technique for use in mass spectrometry‐based proteomics.

### 2.13 Chip‐quantitative polymerase chain reaction (ChIP‐qPCR)

Determining whether the ChIP enhances DNA sequences related to the target protein is essential. The design of primers for quantitative PCR (qPCR) can be used to assess whether or not known genomic binding sites are selectively enriched by immunoprecipitation when, there are known genomic binding sites. The primers for CDH1 and VIM surrounding the transcription start site (TSS) were identified in a literature study, and the UCSC In‐Silico PCR tool was utilized to verify and obtain the amplicon region of both genes (Table 2). The chromatin immunoprecipitation (ChIP) assay was performed using a chromatin immunoprecipitation Kit (Catalog Bes5001, BersinBio Technology) according to manufacturer's instructions. Briefly, DNA was sheared by sonicating the cell lysate, which had been cross‐linked with 1% formaldehyde (Sigma‐Aldrich). Santa Cruz Biotechnology Inc. Anti‐ARID1A (PSG3): sc‐32,761, or normal rabbit IgG (Bes5001, BersinBio Technology), was incubated overnight at 4°C with agitation. Both IP and IgG samples were preabsorbed onto the protein A/G bead mixture. To remove the beads from the samples, they were washed in wash buffer. Then, they were rinsed in water for 15 minutes at 65°C with elution buffer. The magnetic beads were collected on the magnetic stand, and the supernatant was transferred to a new centrifuge tube. The DNA was then purified in accordance with the manufacturer's instructions following reverse cross‐linking. Both the ChIP‐enriched DNA and the input DNA were used as templates for qPCR analysis of CDH1 and VIM promoter regions around the transcription start site (TSS). ChIP enrichment was calculated relative to the input DNA and normalized to the GAPDH level.

**TABLE 2:** The Chip‐qPCR primers and their amplicon region

Gene	Sequence
CDH1	CGCTCAAATACACTCCAGCCtggtgacagtgagatcttatctcaaaagaacaacaaaaaaagaggaatcctttagccccctgagactcagctctgctagcagtcttggtactttgtaaatgacacatctctttgcTCTGCAGTACAAGGGTCAGG
VIM	TGACTTCCACCAGGGTaaaaaccactatcactgagttctATTTTGAAACTACGGACG

*Note*: Red colour indicates primer sequences, and a lower letter indicates the amplicon area of the amplicon sequence.

## 3 RESULTS

### 3.1 Identification of EMT‐associated genes in ARID1A‐proficient and ARID1A‐deficient colon cancer cells

**FIGURE 1:**
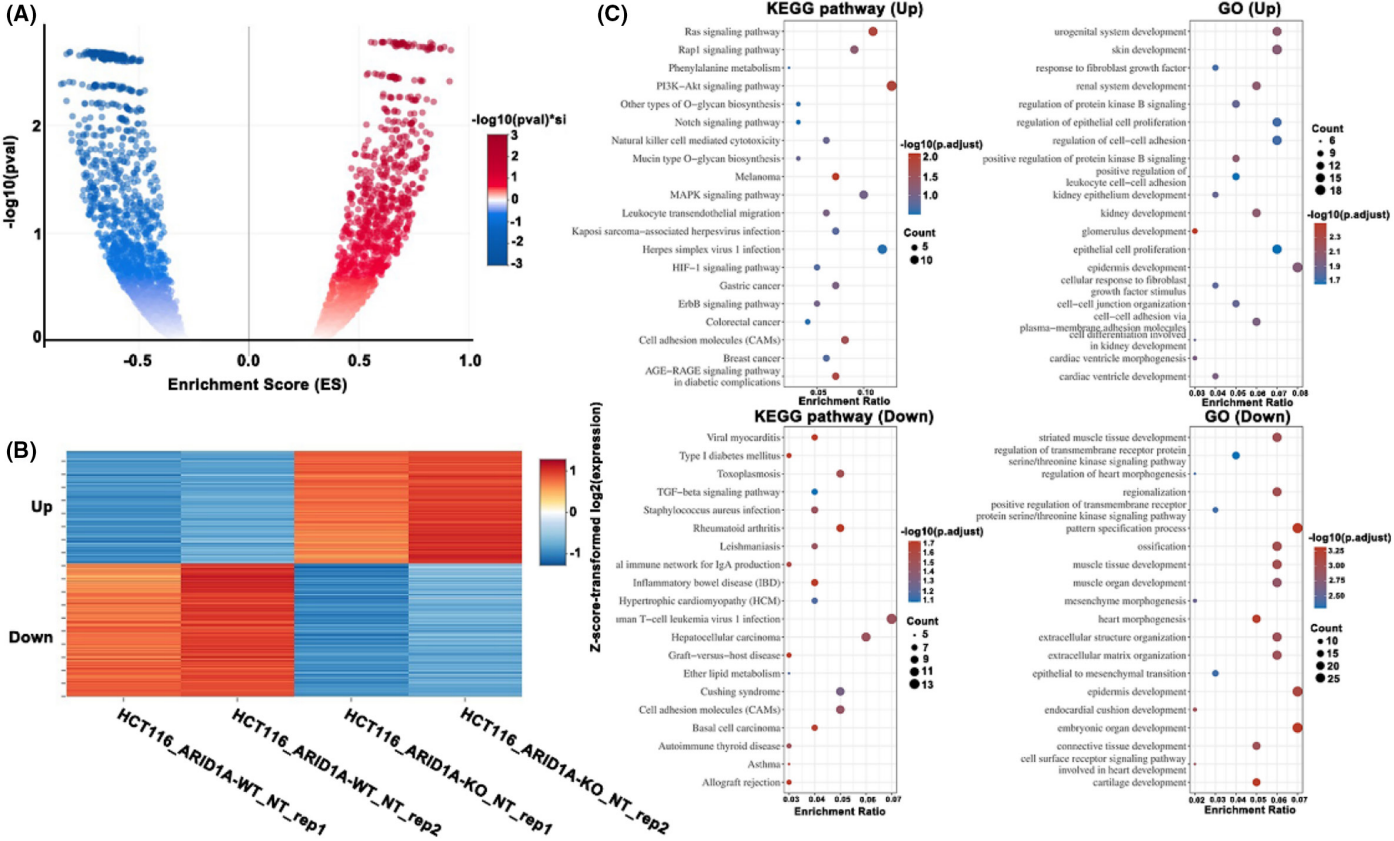
Identified differentially expressed genes (DEGs)in HCT116‐ARID1A‐wild‐type vs. HCT116‐ARID1A‐knockout (A) Volcano plot of differentially expressed genes (B) Heat map of DEGs in control and knockout groups (C) upper part,KEGG and GO enrichment analysis of differentially upregulated genes. (C) lower part ,KEGG and GO enrichment analysis of differentially downregulated genes

We estimated the possible EMT‐associated genes and targets of the ARID1A gene based on the significantly different expression genes in HCT116‐ARID1A Wild type versus HCT116‐ARID1A knocked out cell lines (GSE122926). Using expression cut‐off values of log (fold‐change) > 1.0 or log(fold‐change) < −1.0 and *p*‐value <.05 as cut‐offs, we found 729 differentially expressed genes, including 442 and 287 upregulated and downregulated genes, respectively. The volcano plot (Figure 1A) and heat map (Figure 1B) were built to show the expression of these DEGs in ARID1A‐WT versus HCT116 ARID1A‐KO. The genes identified as a result of ARID1A depletion exhibit a wide range of functional alterations, including EMT (VIM, DLX2, CPA4, FOXC1, WNT11), apoptotic (TGFB1, TGFB2, PRF1), inflammatory (SYK, P2RX7) and inhibitory differentiation function (ID2, ID3). Furthermore, kinases (HSPB8, SYK) and chemokine (CCL26) were included among the identified genes.

### 3.2 ARID1A depletion is involved in cancer‐related signalling pathways in COAD

To better define the ARID1A pathway and related processes, GSVA was used to investigate the expression levels of KEGG pathways. There is an interesting observation because KEGG analyses discovered that genes with increased expression (ARID1A KO vs WT) were implicated in the Ras signalling, Rap signalling, PI3‐AKT signalling, MAPK signalling and colorectal cancer pathways. Whereas genes with decreased expression (ARID1A KO vs WT) were relevant to cancer pathways, including cell adhesion molecules, Type 1 Diabetes Mellitus, toxoplasmosis (Figure 1C). Expectedly, the GO function of upregulated genes associated with ARID1A deficiency has enriched expression in the regulation of epithelial cell proliferation, cell–cell adhesion, epithelial cell proliferation and cell–cell junction organization, as well as in mesenchyme morphogenesis and epithelial to mesenchymal transition (Figure 1C).

### 3.3 Prognostic identification in COAD patients

**FIGURE 2:**
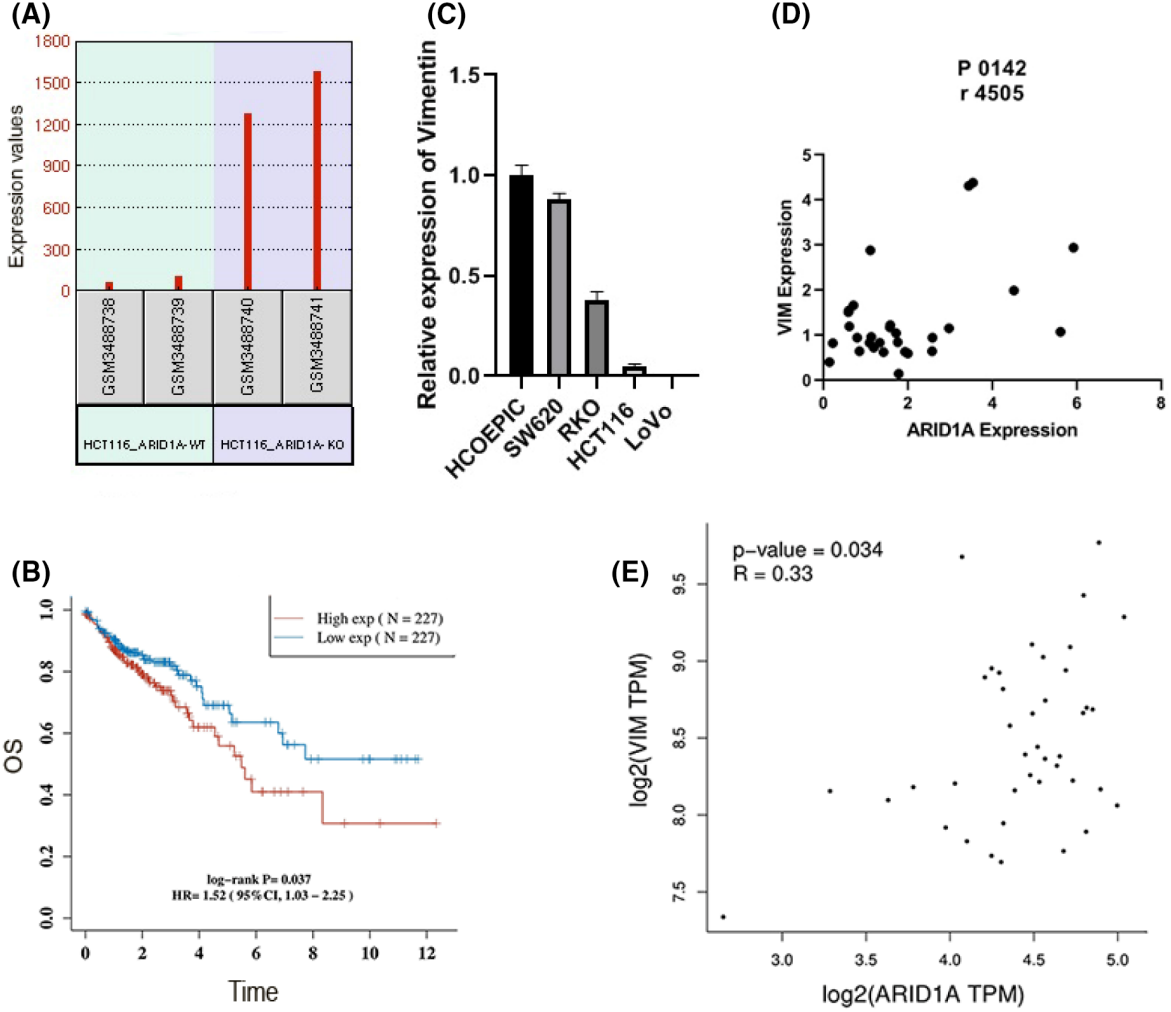
Expression of EMT‐related markers (VIM) and correlation with ARID1A (A) Vimentin (VIM) overexpression was found in KO cell lines (B) VIM high expression is associated with a poor prognosis in COAD (C) VIM displays high expression in cells with lower ARID1A expression (SW620&RKO) and low expression in cells with a high ARID1A expression (HCT116&LoVo) (D) colon cancer samples were examined for co‐expression of Vimentin and ARID1A (E) GEPIA2 database visualization of the co‐expression of Vimentin and ARID1A in colon cancer tissue.

Additional filtering was applied to identify prognostic biomarkers in COAD using survival analysis. Of the 442 upregulated genes identified in the DEGs analysis, 36 genes (Table 3) demonstrated prognostic significance and were associated with a worse prognosis and decreased overall survival. Meanwhile, 12 out of 287 downregulated genes in COAD patients were associated with a poor prognosis and short overall survival. Of these genes, VIM had the highest statistically significant increase in gene expression that is associated with overall survival risk demonstrated by the Kaplan–Meier analysis (*p* = .036810316; HR = 1.520246116; Low 95% CI: 1.025991193; High 95% CI: 2.252600479) (Figure 2A, B).

**TABLE 3:** Upregulated and downregulated genes predicted prognostic significance in COAD.

Genes	*p* Value	HR	Low 95%CI	High 95%CI
VIM	0.03681	1.520246	1.025991	2.2526
GASK1B	0.011975	1.673988	1.12004	2.501905
RUNX1	0.013624	1.647889	1.108121	2.450578
CRIP2	0.017769	1.61103	1.086077	2.389719
CABP1	0.021457	1.59387	1.071321	2.371298
CASTOR1	0.023006	1.586739	1.065709	2.362502
APC2	0.005318	1.76389	1.183437	2.629045
FOXC1	0.028167	1.55552	1.048436	2.30786
CPA4	0.046056	1.494416	1.007057	2.217629
UCP2	0.03854	1.519625	1.022311	2.258863
HSD17B1	0.010328	1.678281	1.129844	2.492934
ITGBL1	0.014706	1.640732	1.102233	2.442315
REEP2	0.040468	1.507296	1.017978	2.23182
PGM5	0.022076	1.587032	1.068656	2.356858
GPRC5A	0.012641	1.65572	1.113969	2.460939
MC1R	0.004736	1.775791	1.192152	2.64516
DNAJB13	0.044492	1.500885	1.010045	2.230251
SLC25A35	0.014369	1.648383	1.104757	2.459513
DLX2	0.007808	1.718028	1.153107	2.559711
ARL4C	0.013519	1.644422	1.108129	2.440262
ESRRB	0.037178	1.518345	1.025131	2.248856
CAPN12	0.018641	1.616113	1.083416	2.410729
AHNAK2	0.017755	1.611121	1.086137	2.389857
PYGM	0.042076	1.510071	1.014871	2.2469
HIST1H2AC	0.018749	1.611751	1.082532	2.39969
ANKRD53	0.01653	1.621629	1.092143	2.407818
TMEM266	0.031602	1.540121	1.038798	2.283381
WNT11	0.016158	1.628241	1.094464	2.422345
FTCD	0.021047	1.589934	1.072268	2.357515
HSPA1L	0.011016	1.676705	1.12566	2.497503
SLC30A4	0.045691	1.496219	1.007747	2.221463
NPAS4	0.017969	1.615591	1.085854	2.403763
WFIKKN1	0.018614	1.605461	1.082319	2.381464
TRIM8	0.039344	1.513212	1.02043	2.243965
TSPAN14	0.048938	1.484553	1.001841	2.199848
HPD	0.033299	1.535194	1.034517	2.278183
**Downregulated genes predicted prognostic significance in COAD**				
MYB	0.006505	0.577725	0.389131	0.857721
GALNT3	0.029541	0.643933	0.433187	0.957208
ARHGAP26	0.023774	0.630414	0.422582	0.94046
MAN1A1	0.032586	0.649939	0.437783	0.964908
ANXA3	0.032062	0.647181	0.434754	0.963403
ATP8B1	0.036793	0.656564	0.442347	0.974521
HENMT1	0.038774	0.657243	0.441423	0.978581
GALNT7	0.047526	0.670787	0.45192	0.995653
ARAP2	0.020746	0.625362	0.420096	0.930925
SYTL2	0.040165	0.659912	0.443683	0.98152
NAT2	0.008401	0.585721	0.393486	0.871872
CCT6B	0.015891	0.613781	0.412774	0.912669

Abbreviation: HR, Hazard ration.

### 3.4 ARID1A significantly correlates with VIM and promotes COAD

Because VIM expression was upregulated in the absence of ARID1A, which was associated with shorter survival in colon cancer patients, we examined VIM expression in colon cancer cell lines and found that VIM expression is high in cells with low ARID1A expression (SW620&RKO) and low in cells with a high ARID1A expression (HCT116&LoVo) (Figure 2C). Thereby, VIM was selected for further analysis. We then examined VIM and ARID1A expression levels in ARID1A COAD tissues to assess ARID1A's correlation with colon cancer. ARID1A significantly correlates with VIM in colon cancer (*p* = 0.0142, *r* = 0.45). Of note, patients with higher ARID1A expression had relatively decreased VIM expression, and conversely, patients with lower ARID1A expression had relatively increased VIM expression, although the correlation was positively significant (Figure 2D). Using GEPIA2, similar results were obtained in ARID1A COAD tumour tissues (*p* = 0.034, *r* = 0.33) (Figure 2E). These findings suggest that ARID1A may influence VIM expression and promote colon cancer.

### 3.5 ARID1A significantly correlates with CDH1 and promotes COAD

**FIGURE 3:**
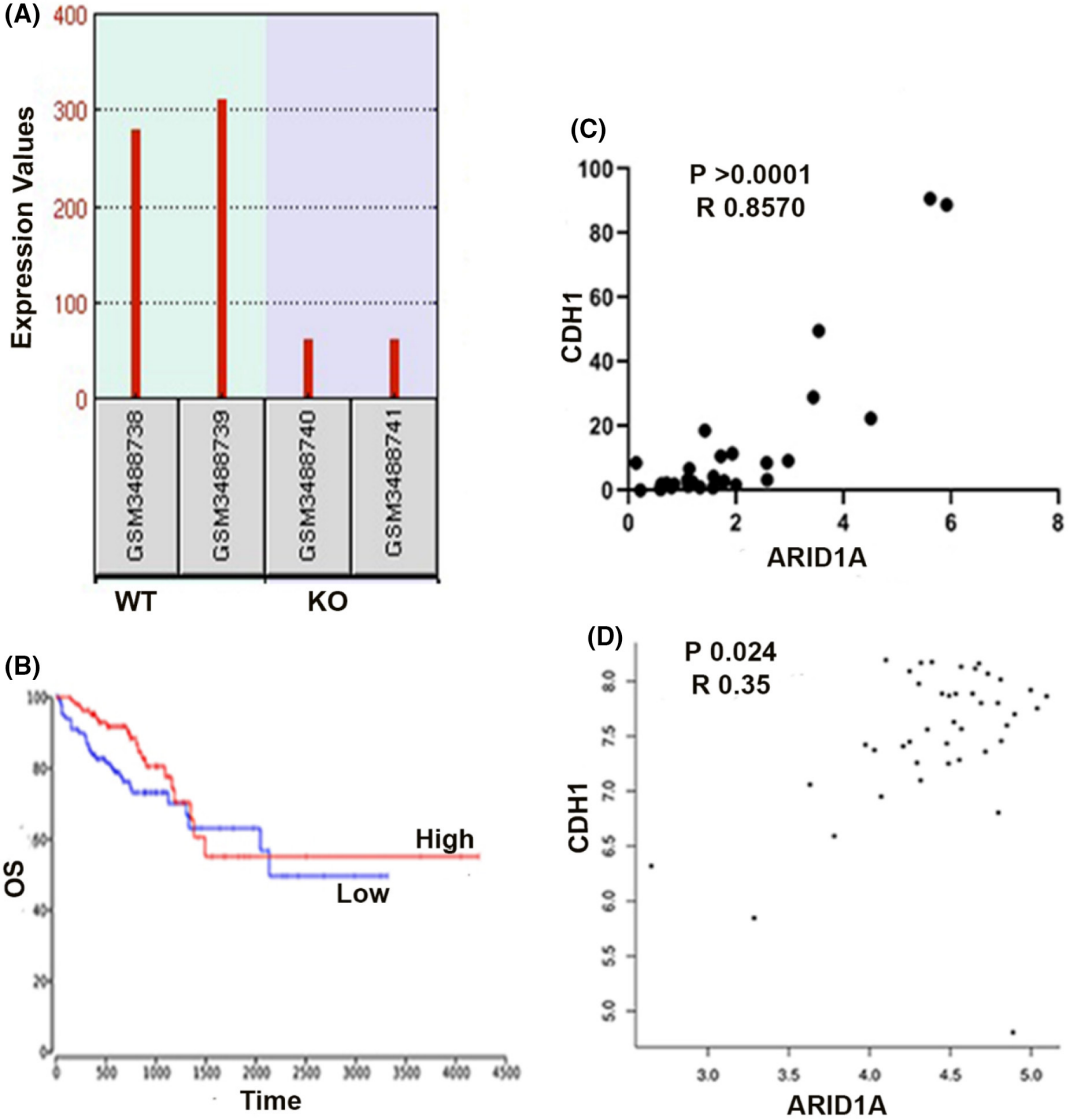
Expression of EMT‐related marker (CDH1) and correlation with ARID1A (A) CDH1 is downregulated in ARID1A deficient cells (B) shows a Kaplan–Maier result of CDH1 in COAD (C and D) E‐cadherin expression correlates positively with ARID1A expression in COAD

Cadherins are well‐studied cell adhesion molecules that have been shown to be tumour suppressors in the laboratory. To better understand ARID1A's role in EMT, we also looked at the possibility of a link between ARID1A and E‐cadherin, a significant epithelial cell adhesion protein that also serves as a tumour suppressor that was found to be downregulated in KO cells when compared to the normal cell group (Figure 3A). CDH1 low expression in colon cancer was associated with short overall survival, although the association was not significant (Figure 3B). The quantitative polymerase chain reaction (qPCR) analysis of ARID1A and E‐cadherin (CDH1) gene expression in patient COAD tumour samples revealed a positive relationship between the two genes regardless of tumour type (Figure 3C). To confirm the relationship between CDH1 and ARID1A expression, we utilized GEPIA2 and investigated CDH1 and ARID1A co‐expression at the mRNA level. Statistically significant of E‐cadherin co‐expression was observed in COAD‐tumour tissues (Figure 3D). These findings imply that loss of ARID1A expression in human tumours affects the levels of E‐cadherin and enhances colon cancer progression.

### 3.6 ARID1A deficiency activates VIM and suppresses CDH1

**FIGURE 4:**
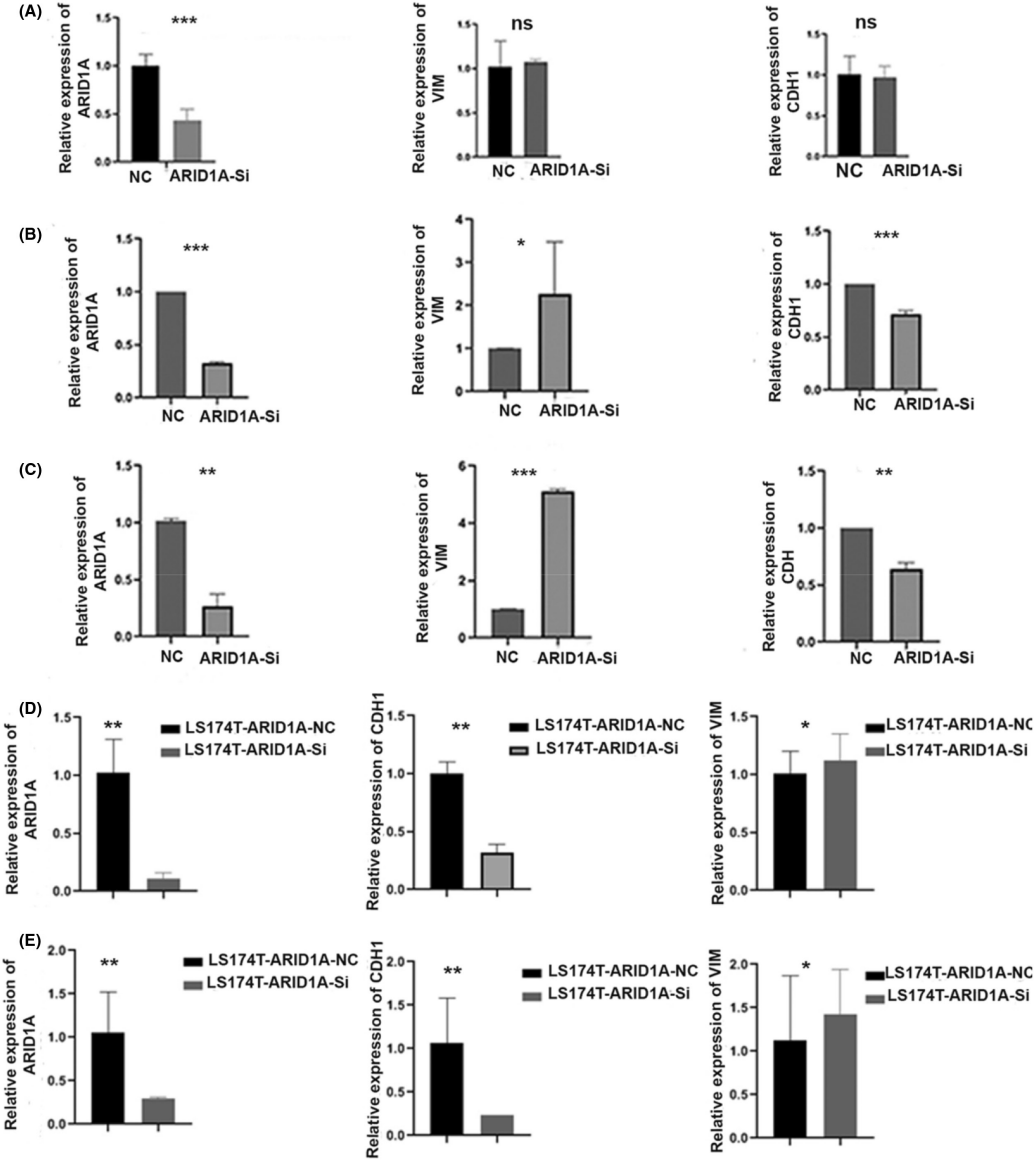
ARID1A knockdown in HCT116 cell line (A) At 50 nM, 4 μl siRNA‐ARID1A, qPCR demonstrates no change of vimentin and E‐cadherin expression levels in HCT116 after 24 h transfection (B) ARID1A deficiency at 75 nM, 6 μl siRNA‐ARID1A for 24 h activates VIM and suppress CDH1 in HCT116 (C) qPCR results of ARID1A downregulation using 75 nM, 6‐μl siRNA for 48 h showed VIM and CDH1 expression in HCT116. ARID1A silencing altered VIM and E‐cadherin expression (D and E) VIM and CDH1 expression were altered in qPCR results of LS174‐T‐ARID1A knocked down for 24 and 48 h, respectively. ns, Non‐significant; *p < 0.05, **p < 0.001, and ***p < 0.0001

HCT‐116, SW480 and LS174T colorectal cell lines containing ARID1A wild type sequence were transfected with siRNA pool to suppress ARID1A expression (Table 4). Notably, Vimentin and CDH1 expression was unchanged when ARID1A was knocked down in HCT116 with 50 nM, 4 μl of siRNA, as verified by qPCR (Figure 4A). We therefore determined the transfection efficiency in time‐dependent manner and a detectable change of VIM and CDH1 expression was observed in HT116‐ARID1A‐knocked down cells using 75 nM, 6 μl siRNA targeting ARID1A after 24 h and 48 h transfection (Figure 4B, C). The LS174T cells were then transfected for further confirmation for 24 and 48 h, and it was discovered that siRNA works very efficiently, giving consistent results (Figure 4D, E). These findings suggest that VIM overexpression, CDH1 lower expression, and metastasis are strongly related to ARID1A depletion in CRC.

**TABLE 4:** ARID1A sequence and mutation profiles in CRC cells used in the research

Cell Name	Primary/Metastasis	Variant classification	Variant Annotation	Variant Type	Protein change	TCGA hotspot count
HCT116	Primary	Non	Non	Non	Non	Non
LoVo	Metastases	Frame_Shift_Del	damaging	DEL	p.P1923fs	16
SW620	Metastases	Non	Non	Non	Non	Non
SW480	Primary	Non	Non	Non	Non	Non
RKO	Primary	Frame_Shift_Del	damaging	DEL	p.P1115fs	3
	Primary	Frame_Shift_Del	damaging	DEL	p.G1632fs	24
LS174T	Primary	Non	Non	Non	Non	Non

**FIGURE 5:**
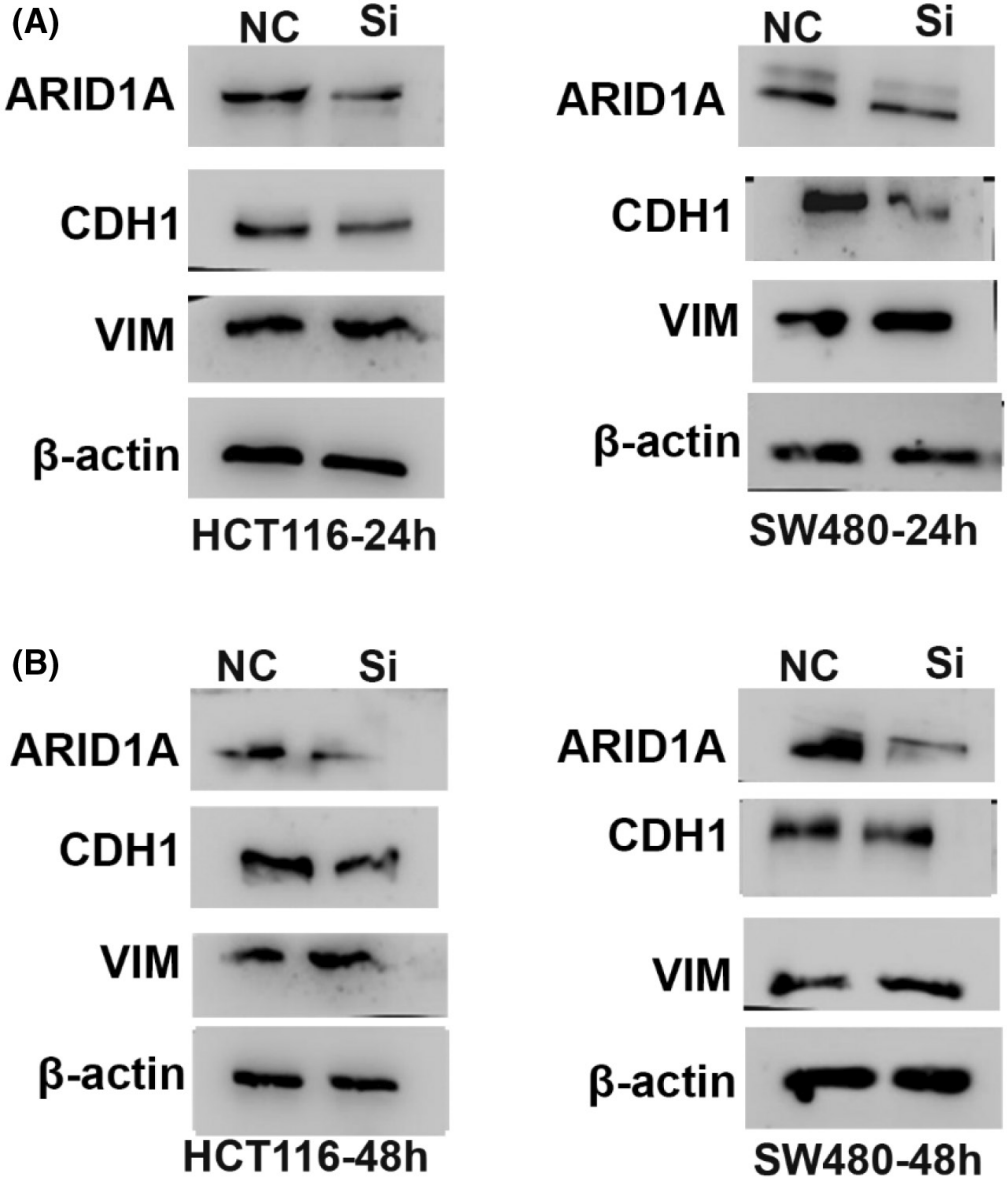
ARID1A silencing alters VIM and E‐cadherin expression (A and B) VIM and CDH1 expression were altered in WB results after ARID1A knocked down for 24 and 48 h.

To further confirm the relationship between CDH1, VIM and ARID1A, we utilized ARID1A knocked down cells (HCT116 and SW480) and analysed their protein expression. Downregulation of CDH1 and upregulation of VIM protein expression was confirmed in the ARID1A‐knocked down cells. ARID1A knockdown efficiency at different concentration and times was confirmed by Western blotting using β‐Actin as an internal control (Figure 5A, B). These findings imply that loss of ARID1A expression in cancer cells in vitro and in human tumours affects the levels of E‐cadherin in cancer cells in vivo.

### 3.7 VIM overexpression activates the EMT pathway

**FIGURE 6:**
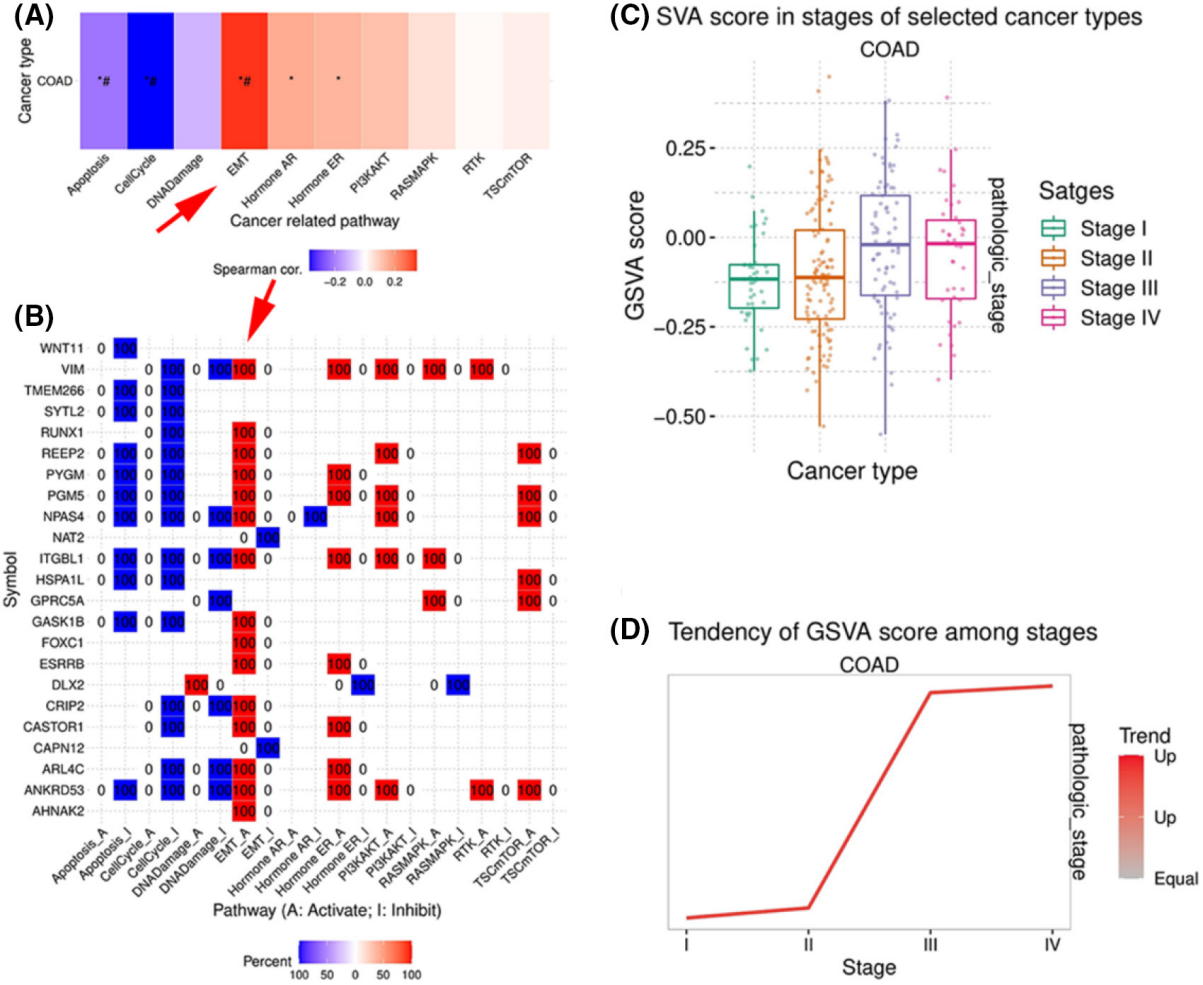
Expression and Pathway activity of upregulated genes in COAD (A) The variations in pathway activity between high and low mRNA expression are shown (B)VIM and other upregulated genes activate EMT pathway in COAD (C) Expression differences between stages are shown (D) Expression tendency in pathological stages from early stage to late stage

Because the epithelial–mesenchymal transition (EMT) is important for cancer growth, invasion and metastasis, we used GSCA to determine gene expression at different stages of colon cancer. The percentage of tumours in which the mRNA expression of DEGs has a metastasis suppressor or activator for colon cancer was determined. GCSA found that most genes were active in the EMT cancer‐related pathways (indicated by red colour). Moreover, at diseased stages, the expression of these genes increased significantly. Noticeably, VIM was among the highest‐ranked genes to activate the EMT pathway (Figure 6), suggesting that ARID1A depletion altered the EMT pathway most profoundly through activating the VIM gene in the colon.

### 3.8 The role of ARID1A loss in colon cancer cell proliferation and migration

**FIGURE 7:**
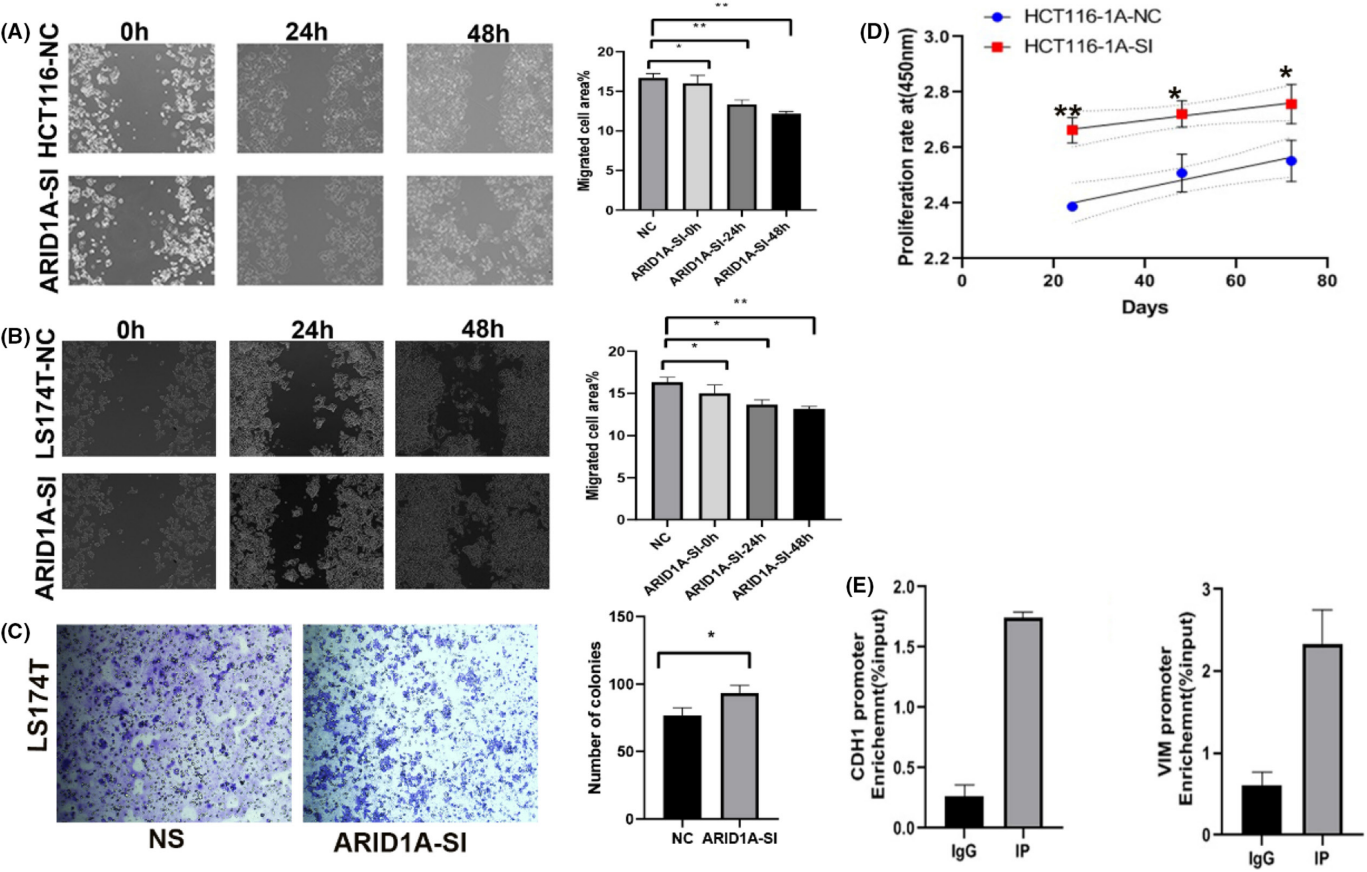
ARID1A knockdown promotes colon cell line migration and proliferation (A and B) The effect of ARID1A depletion on HCT116 and LS174T cell migration (C) The effect of ARID1A depletion on LS174T cell invasion (D) The proliferation of HCT116 cells was assessed at specified times following ARID1A siRNA transfection (E) Immunoprecipitated DNA from ChIP experiments with anti‐ARID1A antibody was examined by real‐time qPCR using E‐cadherin and Vimentin‐specific promoter primers. IP stands for Immunoprecipitated. *p < 0.05, **p < 0.001, and ***p < 0.0001. Statistical differences were calculated using an imageJ software.

Measurement of migration ability indicates that when ARID1A expression is decreased, migration ability increases as well (Figure 7A, B). A transwell experiment was also used to explore the effect of ARID1A silencing on the colon cells. ImageJ software was used to quantify and evaluate digital pictures of migratory cells and wound regions captured with an Olympus microscope (Olympus Inc.). Our findings revealed that ARID1A insufficiency considerably increased the number of migrating cells, as shown in Figure 7C. CCK‐8 assay results showed that the proliferation of HCT116 cell lines was significantly improved following transfection with siARID1A. This is shown in Figure 7D. These findings suggest that ARID1A loss promotes COAD migration, proliferation and colony formation.

### 3.9 ARID1A does not interact with CDH1 or VIM, but rather acts as a TF to regulate their expression in COAD

We aimed to investigate the interaction mechanism between ARID1A and EMT marker genes in CRC cells. Since gene expression levels can be changed either transcriptionally or post‐translationally, we first utilized co‐IP to identify ARID1A novel interacting proteins and determine whether ARID1A could interact with VIM and CDH1 to regulate their expression in the colon cell line. The HCT116 cells expressing ARID1A wild type were harvested and utilized for Co‐IP with Co‐IP Kit. Samples were prepared using the in‐gel digestion method, a mass spectrometry‐based technique. As a result, a wide range of novel proteins was observed to interact with ARID1A (Table 5). However, ARID1A did not co‐precipitate with CDH1 or VIM in HCT116 cells. The proteins of ARID1A and IgG were located using a Venn diagram, and ARID1A was shown to interact with 87 different proteins. Given that ARID1A contributes to gene activation or repression via nucleosome position remodelling, it is possible that ARID1A directly represses VIM transcription by occupying its promoter. Therefore, we performed chromatin immunoprecipitation (ChIP) studies on the VIM and CDH1 promoters. Our ChIP experiments on the VIM and CDH1 promoters utilized specific antibodies against ARID1A and IgG as well as a primer pair that targeted the transcription start site (TSS) of both genes. Indeed, the ChIP experiments with an anti‐ARID1A antibody greatly enriched the VIM and CDH1 promoter areas (Figure 7E).

**TABLE 5:** List of 87 ARID1A interaction proteins in HCT116

UniProt Accession	Gene Symbol	UniProt Accession	Gene Symbol	UniProt Accession	Gene Symbol	UniProt Accession	Gene Symbol
Q969G3	SMARCE1	Q02383	SEMG2	O43809	NUDT21	Q9HD64	XAGE1A XAGE1B
P00167	CYB5A	P11279	LAMP1	P62136	PPP1CA	O75635	SERPINB7
Q14315	FLNC	P0DOX8	IGL1	Q12840	KIF5A	P62304	SNRPE
Q9NYF8	BCLAF1	Q9UKG1	APPL1	Q5TEC6	H3‐2	P63267	ACTG2
P61513	RPL37A	Q6UWP8	SBSN	A0A087WW87	IGKV2‐40	Q14533	KRT81
O14497	ARID1A	Q99578	RIT2	P36894	BMPR1A	Q14247	CTTN
P63218	GNG5	Q5QNW6	H2BC18	P60059	SEC61G	O15231	ZNF185
Q9HD42	CHMP1A	P51532	SMARCA4	Q12792	TWF1	P49862	KLK7
Q53RT3	ASPRV1	Q6BDS2	UHRF1BP1	Q8TDY2	RB1CC1	P62891	RPL39
Q8NFJ5	GPRC5A	O96019	ACTL6A	Q8WVV4	POF1B	Q8N684	CPSF7
Q9NR12	PDLIM7	Q8TAA3	PSMA8	P15531	NME1	Q8TAQ2	SMARCC2
P02647	APOA1	P30044	PRDX5	P23246	SFPQ	Q00534	CDK6
O75342	ALOX12B	Q8TCV5	WFDC5	P01042	KNG1	Q71UI9	H2AZ2
P17096	HMGA1	O75676	RPS6KA4	A8MTJ3	GNAT3	Q8N264	ARHGAP24
Q7Z3Y8	KRT27	P56385	ATP5ME	P01859	IGHG2	P07910	HNRNPC
Q14CN4	KRT72	Q9UG63	ABCF2	A8MPP1	DDX11L8	P62888	RPL30
Q86VE3	SATL1	P19013	KRT4	Q9BQE3	TUBA1C	P05164	MPO
Q9P0K7	RAI14	Q16630	CPSF6	Q9P219	CCDC88C	P15954	COX7C
P60900	PSMA6	P16989	YBX3	P30626	SRI	Q14532	KRT32
Q6P5R6	RPL22L1	Q9H444	CHMP4B	P42167	TMPO	Q15388	TOMM20
O75717	WDHD1	Q9NX63	CHCHD3	Q99935	OPRPN	P25398	RPS12
P80188	LCN2	A8K2U0	A2ML1	O15144	ARPC2		

*Note*: Results were obtained from HCT116 using CO‐IP.

## 4 DISCUSSION

The SWI/SNF chromatin remodelling complex is crucial in regulating gene expression through epigenetic mechanisms.^21^ In addition to its DNA‐binding domain, ARID1A is an essential component of the SWI/SNF complex, as it is responsible for targeting the complex to gene promoters. By repositioning nucleosomes, the ARID1A and SWI/SNF complexes enable the activation or repression of transcription of hundreds of target genes, including p21 (CDKN1A), SMAD3 (a molecular mediator of stress‐induced cellular proliferation), and THBS1 (which modulates telomere length), as well as TERT (a key component of telomerase reverse transcriptase).^5^ In patients with gastric carcinoma, decreased ARID1A expression was associated with lymph node metastases, tumour infiltration and poor prognosis.^13^ Similarly, our prior study revealed that lower ARID1A expression was associated with lymph node and distant metastases, as well as a poor prognosis in COAD patients.^12^ Here, colon cancer and colon development‐associated gene sets were particularly abundant in the ARID1A upregulated group.

In many cancers, tumour development and metastasis are connected with increased Vimentin expression and reduced E‐cadherin expression.^22^, ^23^, ^24^ Because EMT is a crucial step in colon cancer growth, invasion and metastasis,^25^, ^26^ we searched for EMT‐associated genes in ARID1A knockdown cells and showed that ARID1A deficiency leads to EMT phenotype and affects the survival of COAD patients via targeting EMT genes. The formation of an EMT phenotype occurs when EMT‐related markers, such as Vimentin and fibronectin, are increased and other adhesion factors, such as E‐cadherin, are decreased.^27^ The vimentin gene has a crucial role in regulating EMT through epithelial marker reduction and mesenchymal marker overexpression.^28^ This study demonstrated that high vimentin expression was significantly associated with ARID1A decreased expression and predicted poor prognosis in COAD. In colorectal cancer, TRIM29‐overexpressed cells also showed VIM activation contributing to migration, invasion and metastases.^29^ ARID1A knocking down promotes cell migration in COAD cells via VIM upregulation, considering that VIM is crucial for migration and invasion in COAD.

E‐cadherin is a cell–cell adhesion core protein that is crucial to maintaining epithelial cell integrity. Our analysis shows a high correlation between the expression of E‐cadherin and ARID1A, suggesting that both interact in some way to govern migration and invasion. We discovered that decreased ARID1A expression suppresses E‐cadherin expression, consequently loosening cell–cell junctions in COAD tissues and promoting tumour cell migration and invasion. These findings corroborate prior findings by He Fei et al,^17^ who reported a positive correlation of ARID1A and E‐cadherin in hepatocellular carcinoma. E‐cadherin expression has been demonstrated to be repressed by an EMT‐induced transcription factor known as ZEB1, which binds to E‐boxes close to the E‐cadherin promoter.^30^, ^31^, ^32^ Recent research has indicated that ARID1A plays a significant function in colon cancer cell proliferation and migration via CDH1 regulation.^33^ Collectively, loss expression of CDH1 and increased expression of VIM appear to contribute to the formation of metastasis via ARID1A loss of expression.^34^ Furthermore, the cellular enrichment analysis of highly elevated and downregulated genes showed considerable evidence of cell–cell adhesion and mesenchymal epithelial transformation. Notably, the decreased Vimentin expression in HCT116 with ARID1A wild type rather than ARID1A‐mutated CRC cell lines was obvious. E‐cadherin is responsible for processes like cell–cell adhesion, motility, epithelial cell proliferation and serves as an invasive inhibitor.^35^ For the first time, we have found evidence of the ARID1A pathway influencing EMT in colon cancer. Both VIM and CDH1 were identified as target genes for ARID1A in mechanism activity and exhibited higher enrichment Chip‐qPCR utilizing anti‐ARID1A antibody.^36^ ARID1A's ability to influence EMT via other molecular mechanisms remains an open question for future studies. In conclusion, CRC cells with ARID1A silencing had higher levels of VIM and lower levels of CDH1 expression, which are both positively correlated with ARID1A in colon cancer tissues.^37^ The in vitro migration and invasion of CRC cells are accelerated by the reduction of ARID1A expression. VIM and CDH1 appear to be ARID1A's molecular targets in colon cancer, which improve colon cancer cell viability, invasion and proliferation, especially in cells with ARID1A downregulation. Thus, ARID1A represents a molecular‐targeted treatment for COAD.

## AUTHOR CONTRIBUTIONS

**Salem Baldi:** Conceptualization (lead); data curation (lead); formal analysis (lead); investigation (equal); methodology (equal); resources (equal); software (equal); validation (equal); writing – original draft (lead); writing – review and editing (equal). **Qianshi Zhang:** Resources (equal). **Zhenyu Zhang:** Methodology (equal). **Mohammed Safi:** Resources (equal). **Hassan Khamgan:** Validation (equal). **Han Wu:** Methodology (equal). **Mengyan Zhang:** Methodology (equal). **Yuanyuan Qiian:** Methodology (equal). **Yina Gao:** Methodology (equal). **Abdullah Shopit:** Methodology (equal). **Abdullah Aldanakh:** Formal analysis (equal). **Mohammed Alradhi:** Software (equal). **Murad Al Nusaif:** Software (equal).

## CONFLICT OF INTEREST

The authors disclose no potential conflicts of interest.

## CONSENT FOR PUBLICATION

All patients provided written consent for publication.

## Data Availability

The datasets generated during and/or analysed during the current study are available in the following links: Home ‐ GEO ‐ NCBI (nih.gov) (GSE122926) http://www.home‐for‐researchers.com/static/index.html#/ GEPIA 2 (cancer‐pku.cn) http://bioinfo.life.hust.edu.cn/GSCA/#/ DepMap: The Cancer Dependency Map Project at Broad Institute Cell line LS174T ‐ Mutations | canSAR Black (icr.ac.uk)
