# Correction to ‘ARID1A downregulation promotes cell proliferation and migration of colon cancer via VIM activation and CDH1 suppression’

**DOI:** 10.1111/jcmm.70191

**Published:** 2025-02-11

Baldi S, Zhang Q, Zhang Z, et al. ARID1A downregulation promotes cell proliferation and migration of colon cancer via VIM activation and CDH1 suppression. *J Cell Mol Med.* 2022;26:5984‐5997. doi:10.1111/jcmm.17590.

In Salem Baldi et al., the VIM image of HCT116 cells in Figure 5B overlapped the VIM in Figure 5A due to technical error during image preparation. The correct figure is shown below. The authors confirm all results and conclusions of this article remain unchanged.

**FIGURE 5:**
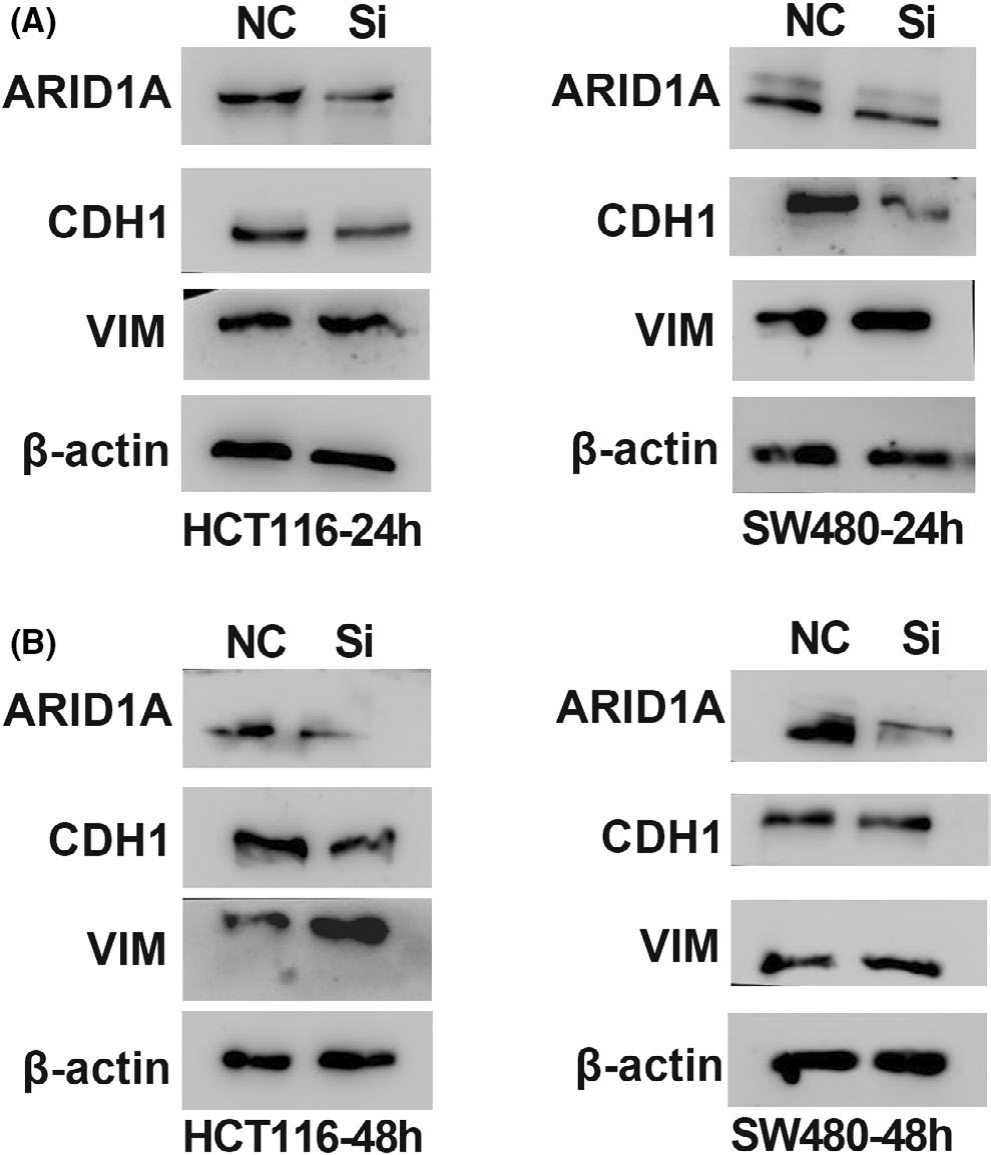
ARID1A silencing alters VIM and E‐cadherin expression (A and B) VIM and CDH1 expression were altered in WB results after ARID1A knocked down for 24 and 48 h.

