# Correction to “ARID1A Downregulation Promotes Cell Proliferation and Migration of Colon Cancer via VIM Activation and CDH1 Suppression”

**DOI:** 10.1111/jcmm.70381

**Published:** 2025-05-01

S. Baldi, Q. Zhang, Z. Zhang, et al., “ARID1A Downregulation Promotes Cell Proliferation and Migration of Colon Cancer via VIM Activation and CDH1 Suppression,” *Journal of Cellular and Molecular Medicine* 26 (2022): 5984–5997, https://doi.org/10.1111/jcmm.17590.

In Salem Baldi et al., several incorrect images were used in the published version. They include: The VIM image of HCT116 cells in Figure 5B, ARID1A‐SI of 0 h in Figure 7B. The correct figures are shown below. The authors confirm all results and conclusions of this article remain unchanged.

**FIGURE 5:**
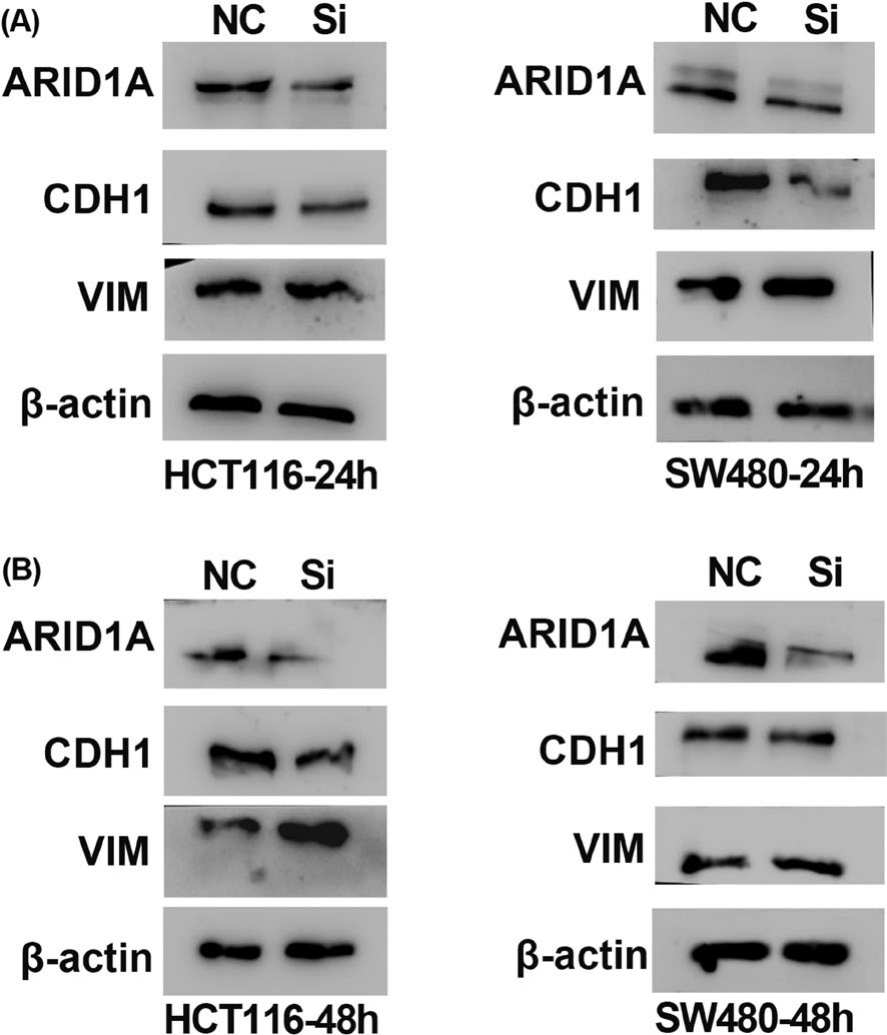
ARID1A silencing alters VIM and E‐cadherin expression (A and B) VIM and CDH1 expression were altered in WB results after ARID1A knocked down for 24 and 48 h.

**FIGURE 7:**
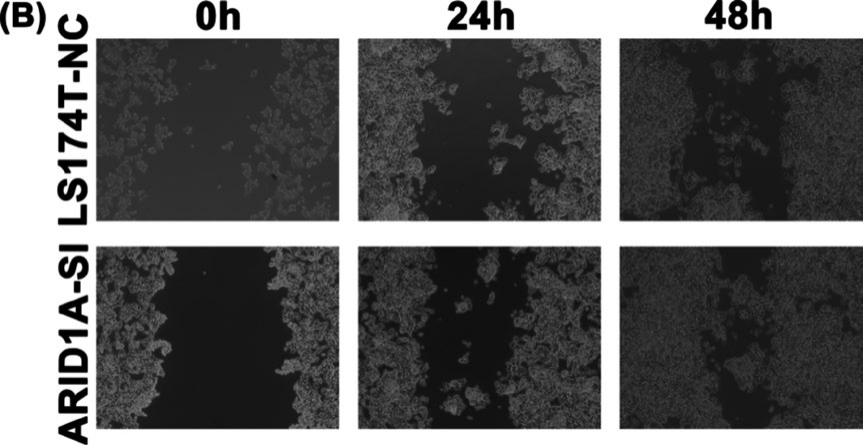
ARID1A knockdown promotes colon cell line migration and proliferation (B) The effect of ARID1A depletion on LS174T cell migration.

